# A new risk-stratified scoring system for predicting left atrial appendage thrombus in patients with nonvalvular atrial fibrillation

**DOI:** 10.1186/s12872-025-05348-6

**Published:** 2025-12-20

**Authors:** Junhao Liu, Xuefeng Zhu, Xiaobo Zheng, Mengmeng Ren, Yanyan Jing, Hongxia Chu

**Affiliations:** 1 School of Clinical Medicine, Shandong Second Medical University, Baotong West Street, Weicheng District, Weifang City, Shandong 261053 China; 2 Department of Cardiology, The affiliated Yantai Yuhuangding Hospital of Qingdao University, Yantai, Shandong 264000 P. R. China; 3 Qingdao Central Hospital, University of Health and Rehabilitation Sciences (Qingdao Central Medical Group), Qingdao, 266042 China

**Keywords:** Left atrial appendage thrombus, Atrial fibrillation, Transesophageal echocardiography

## Abstract

**Background:**

The predictive capacity of the CHA2DS2-VASc score for left atrial appendage thrombus (LAAT) detection in nonvalvular atrial fibrillation (NVAF) patients shows significant limitations.

**Objective:**

To recognize predictors of LAAT and create a more precise risk-assessment model.

**Methods:**

Between January 2019 and December 2023, consecutive NVAF patients without a previous history of anticoagulation who underwent transesophageal echocardiography were recruited from two centers. Clinical data, biomarker information, and transthoracic echocardiographic parameters were collected in a standardized manner. The derivation cohort comprised patients from one center, while the validation cohort consisted of participants from the other center.

**Results:**

1505 patients (mean age 63.4±9.77 years; 895 male) were analyzed. LAAT was detected in 109 (7.24%) of the total 1505 patients. The final parameters selected for the LEFT-AF risk model included left atrial diameter (LAD), left ventricular ejection fraction (LVEF), history of heart failure (HF), history of stroke, and non-paroxysmal AF (NPAF). In the derivation cohort, the novel scoring system demonstrated superior discriminative performance compared to the currently used CHA2DS2-VASc score (0.693, 95% CI 0.634-0.752) (P<0.001), CHA2DS2 score (0.679, 95% CI 0.621-0.731) (P<0.001) and CLOTS-AF score (Creatinine>1.5mg/dL, LVEF<50%, LAVI>34ml/m2, TAPSE<17mm, Stroke, AF rhythm) (0.762, 95% CI 0.704-0.821), with an area under the receiver operating characteristic curve (AUC) of 0.855 (95% CI 0.808-0.902) (P<0.001). This same superior performance was maintained in the validation cohort.

**Conclusion:**

Among patients with NVAF who had not previously received anticoagulation therapy, the prevalence of LAAT was 7.24%. The LEFT-AF score improves LAAT risk stratification, particularly in patients with low CHA2DS2-VASc scores, potentially guiding anticoagulation decisions.

**Graphical abstract:**

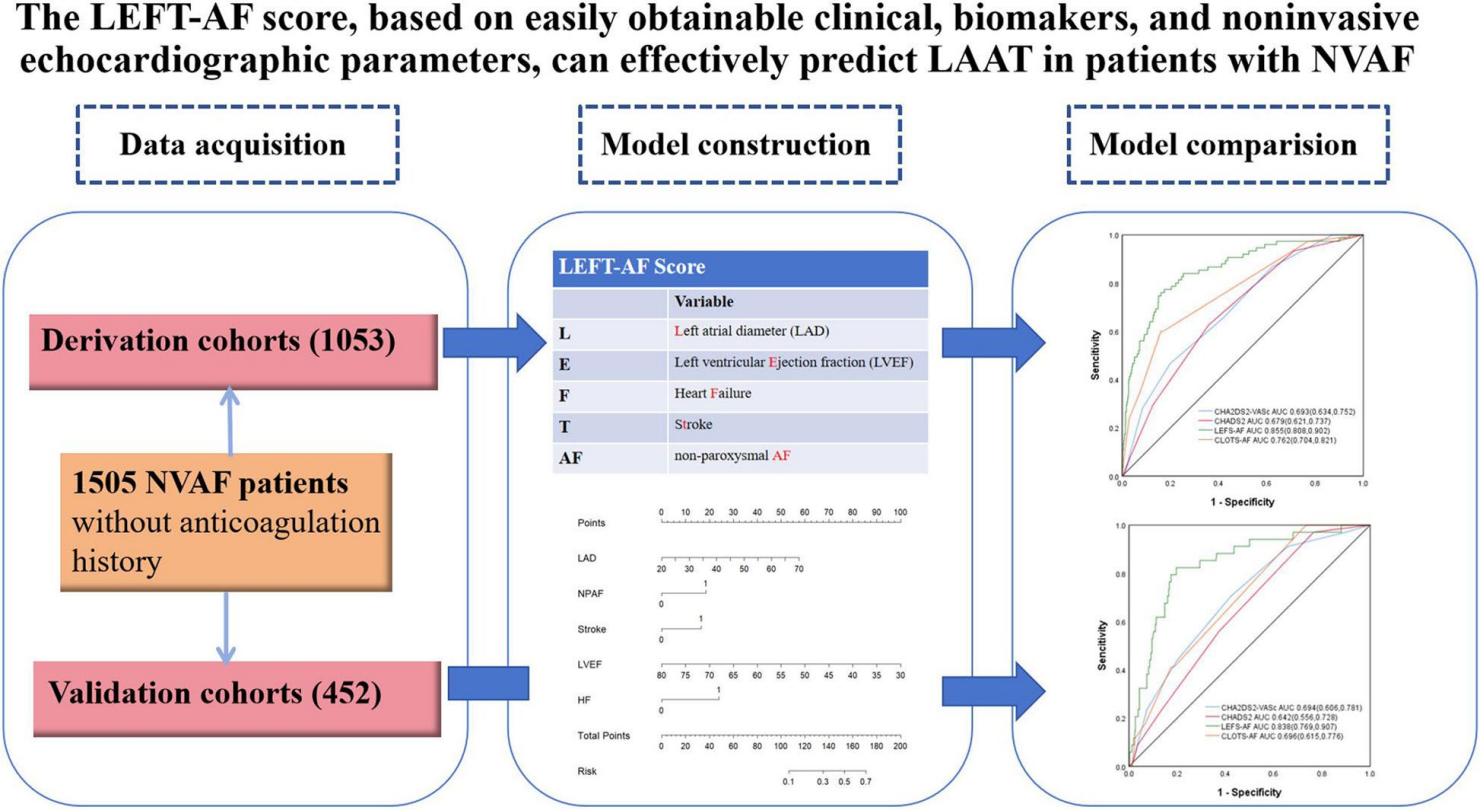

**Supplementary Information:**

The online version contains supplementary material available at 10.1186/s12872-025-05348-6.

## Introduction

One of the contributing factors to thromboembolic stroke is atrial fibrillation (AF). Autopsy and surgical findings have shown that, in patients with non-valvular atrial fibrillation (NVAF), 90% of atrial thrombi originate from the left atrial appendage [1]. Contemporary clinical guidelines for stroke prophylaxis in NVAF predominantly utilize the CHADS2 and CHA2DS2-VASc scores [2]. Nevertheless, the predictive accuracy of these scoring systems for left atrial appendage thrombus (LAAT) in NVAF remains limited [3–6]. One possible reason for the suboptimal predictive values is that the CHA2DS2-VASc score assigns equivalent weight to most risk factors without additional stratification. Moreover, recent studies suggest that when no accompanying thromboembolic risk factors are present, the higher scores assigned to female factors in the CHA2DS2-VASc score should be seen as risk modifiers for AF, rather than a standalone risk factor [7, 8]. Another reason for the reduced predictive performance is that the CHA2DS2-VASc score relies solely on clinical parameters for risk assessment and fails to incorporate other risk factors associated with a significant increase in LAAT, as revealed by recent research [9–12]. 

Numerous studies have attempted to develop various predictive models using clinical, biomarker, and/or transesophageal echocardiography parameters to predict the occurrence of stroke and LAAT [13–17]. However, due to the variability of predictors incorporated into these models across different studies, there is a lack of widely accepted risk schemes for clinical application. Thus, the objective of this study was to try to create a new, more elaborate risk stratification scoring system.

## Methods

### Study population

This retrospective, two-centers study involved 3196 consecutive patients with AF who underwent transesophageal echocardiography (TEE) between January 2019 and December 2023 for the detection of LAAT before cardioversion or catheter ablation. We excluded patients who underwent TEE for reasons unrelated to AF, those with a history of rheumatic heart disease, prosthetic valve placement, or current oral anticoagulation therapy, and eliminated duplicate studies to establish the final research cohort. The clinical, echocardiographic, and laboratory examination data were systematically extracted from electronic health records. For patients with multiple TEE examinations, only the initial TEE results were involved in the analysis. The type of AF was classified according to widely accepted guidelines [18]. Patients with persistent or ‘permanent’ AF were classified as having non-paroxysmal AF (NPAF). All participants were assigned a CHA2DS2-VASc score based on established guideline criteria [19]. The study was approved by the institutional ethics review board at Qingdao University Affiliated Yantai Yuhuangding Hospital and Qingdao Central Hospital, University of Health and Rehabilitation Sciences, and conducted according to Declaration of Helsinki. All participants were informed of the study’s purpose, and written informed consent was obtained.

### Transthoracic and transesophageal echocardiography

As part of their cardiological assessment before TEE, all patients underwent comprehensive 2D TEE performed by accredited cardiac sonographers. Available equipment (Vivid 7 or E9, GE Medical Systems) was used for both transthoracic and transesophageal echocardiography. For transthoracic echocardiography, a 2.5- or 3.5- MHz phased-array transducer was used. The retrospective collected echocardiographic data involved the measurement of left atrial diameter (LAD) and left ventricular end-diastolic diameter (LVEDD) in the parasternal long-axis view during late diastole. Additionally, the left ventricular ejection fraction (LVEF) was calculated using Simpson's formula. This calculation utilized 2-dimensional images of the left ventricle chamber in systole and diastole from the 4- and 2-chamber apical views. Standard tomographic planes were obtained during the TEE examination. A thrombus was defined as a circumscribed, uniformly echo-dense mass distinguishable from the underlying left atrial endocardium and pectinate muscles [20], spontaneous echo contrast (SEC) was excluded from the assessment. When LAAT was suspected, two echocardiographers evaluated the study. In cases where there was doubt, a third echocardiographer was involved to reach a unanimous and most reliable diagnosis.The treating physician had the discretion to decide whether to adjust anticoagulation after a diagnosis of LAAT. For a subset of patients with LAAT, the treating physician determined that they would undergo repeat TEE imaging at least 6 weeks later. Our objective was to find out the clinical and imaging features related to thrombus resolution in this subgroup. Thrombus resolution was defined as the non - detection of any thrombus on TEE.

### Statistical analysis

For normally distributed quantitative variables, means and standard deviations were calculated, and unpaired Student's t-tests were performed. Non-normal distributions were described using interquartile ranges and analyzed via Mann-Whitney tests. Categorical variables were expressed as counts with percentages and compared utilizing Chi-square tests or Fisher’s exact tests. Statistical significance was defined as a two-tailed P value below 0.05. In this study, consecutive patients from Yantai Yuhuangding Hospital Affiliated to Qingdao University were enrolled in the derivation cohort for the development of the LEFT-AF score. Simultaneously, consecutive patients from Qingdao Central Hospital, University of Health and Rehabilitation Sciences, were incorporated into the external validation cohort to independently assess the performance of the model. The predictive scoring system was developed through the following steps: patients in the derivation cohort were stratified into LAAT and non-LAAT groups based on TEE findings, and a lasso regression model was applied to reduce multicollinearity. Univariate and multivariate logistic regression analysis was performed based on the variables retained by lasso regression. Through multivariate logistic regression analysis, a nomogram model was constructed. Subsequently, its diagnostic performance was evaluated with area under the receiver operating characteristics curve (AUC) values. To assess incremental discrimination beyond existing criteria, we used Delong’s test to compare the AUC values from our derived score system with CHA2DS2-VASc , CHADS2 and CLOTS-AF score systems [16]. The Hosmer-Lemeshow goodness-of-fit test (*P*≥0.05) was employed to assess model calibration in the validation cohort. A separate validation cohort was employed to assess the scoring system’s performance. Statistical computations were performed with SPSS 26.0 and R 4.3.2.

## Results

### Baseline characteristics

3196 patients undergoing TEE were evaluated for eligibility. 1691 patients were excluded. Regarding the exclusions, 1066 patients had a history of using anticoagulant drugs, and 131 patients had a history of rheumatic heart disease or prosthetic valve implantation. Additionally, 107 patients underwent TEE for reasons unrelated to the study's objective, and 287 patients had TEE prior to atrial flutter ablation.

**Fig. 1:**
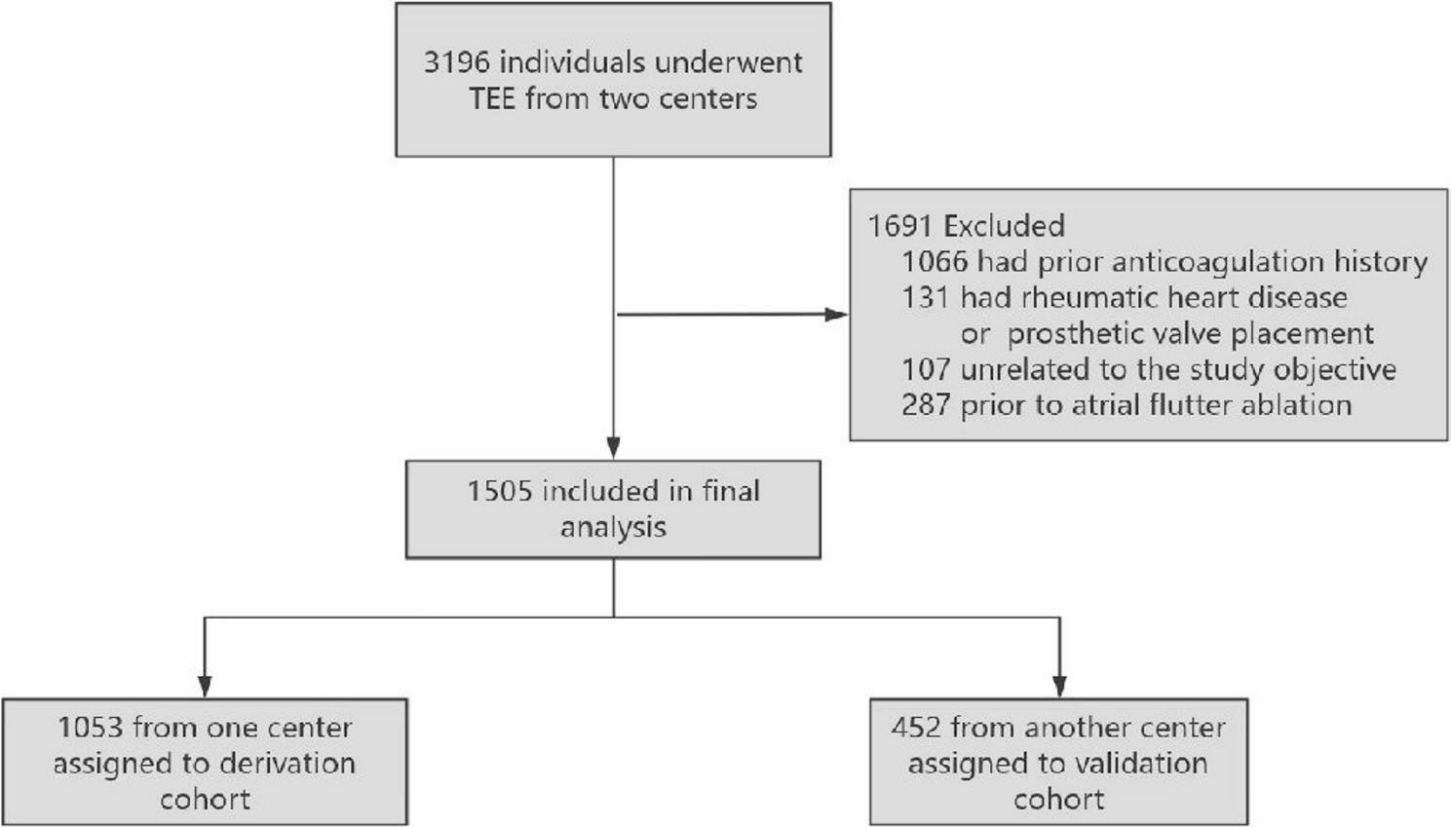
Flow chart of this study

The final analytical cohort consisted of 1505 patients (mean age 63.4±9.77 years; 895 male) who met the diagnostic criteria for NVAF (Fig. 1). Patients from Qingdao University Affiliated Yantai Yuhuangding Hospital were assigned to the derivation cohort, whereas those from Qingdao Central Hospital, University of Health and Rehabilitation Sciences were allocated to the external validation cohort. The baseline characteristics of patients in both the derivation and validation cohorts are presented in Table S1. The baseline characteristics of the two cohorts exhibited substantial comparability. No significant disparities were detected in demographic or clinical variables, indicating that the cohorts were appropriately balanced for external validation.

### Derivation cohort characteristics

The derivation cohort consisted of 1053 patients (mean age 63.38±9.69 years; 633 male). Fig. S1A shows the prevalence distribution of the CHA2DS2-VASc score. The majority of participants were classified as low-risk (12.06% and 24.03% had score of 0 and 1, respectively). LAAT was detected in 75 patients (7.12%). LAAT were detected in 13.33% of patients with a CHA2DS2-VASc score of 1, and in 21.33% of patients with a CHA2DS2-VASc score of 2 or more. Fig. S1B illustrates the proportional distribution of LAAT prevalence, stratified by CHA2DS2-VASc score.

### Predictors of LAAT in the derivation cohort

The comparisons of clinical, biomarkers, and echocardiographic characteristics between patients with and without LAAT are summarized in Table 1. The Lasso regression model, developed usin baseline data, is presented in Fig. S2A and S2B. The results indicate that the model keeps 7 indicators, which are regarded as relatively optimal: LAD, LVEDD, LVEF, Prior stroke or transient ischemic attack (TIA), NPAF, Heart failure (HF) and creatinine. Based on these 7 indicators, univariate and multivariate logistic regression analyses were carried out. Eventually, 5 predictors of LAAT were selected and retained, including LAD (OR, 1.05; P=0.049), LVEF (OR, 0.92; P<0.001), prior stroke/TIA (OR, 2.36; P=0.029), NPAF (OR, 2.35; P=0.020) and HF (OR, 2.99; P<0.001) (Table 2). LAD and LVEF, as continuous variables, are utilized to fully utilize all the information they contain. In the derivation cohort, after multivariate adjustment, the variables that were significantly correlated with left atrial appendage thrombus indicated that a CHA2DS2-VASc score of ≥2 could not independently predict left atrial appendage thrombus [P = 0.169, odds ratio (95% confidence interval): 1.609 (0.818, 3.167)]. 

**Table 1:** Comparison of the baseline characteristics between patients with and without LAAT in the derivation cohort

Variable	NO LAAT(*n* = 978)	LAAT(*n* = 75)	χ^2^/t/z	*P*
Gender			4.758	0.029
Female (%)	399(40.8%)	21(28.0%)		
Male (%)	579(59.2%)	54(72.0%)		
Age (years)	63.46 ± 9.71	62.41 ± 9.43	0.901	0.368
Weight(Kg)	75.5 ± 12.81	77.8 ± 13.31	1.345	0.179
AF type			38.724	< 0.001
Paroxysmal AF(%)	508(51.9)	11(14.7)		
Non-Paroxysmal AF(%)	470(48.1)	64(85.3)		
Duration of Disease(month)	12.00(2.00,60.00)	24.00(3.00,60.00)	0.615	0.538
Drinking(%)	300(30.7)	22(29.3)	0.059	0.808
Smoking(%)	351(35.9)	29(38.7)	0.233	0.629
Heart failure(%)	249(25.5)	55(73.3)	77.742	< 0.001
Hypertension(%)	537(54.9)	44(58.7)	0.398	0.528
Diabetes(%)	195(19.9)	16(21.3)	0.085	0.771
Prior stroke/TIA(%)	63(6.4)	12(16.0)	9.620	0.002
Coronary heart disease(%)	213(21.8)	15(20.0)	0.130	0.718
Other arterial embolism(%)	18(1.8)	5(6.7)	5.503	0.019
DVT(%)	9(0.9)	2(2.7)	--	0.182
COPD(%)	11(1.1)	2(2.7)	--	0.235
MPV	10.04 ± 0.98	10.29 ± 0.92	2.453	0.014
LDL-C	2.91 ± 1.07	2.93 ± 1.01	0.519	0.604
LAD	41.14 ± 6.39	46.12 ± 5.98	6.526	< 0.001
TAPSE	21.78 ± 3.23	19.91 ± 4.05	3.902	< 0.001
LVEDD	45.78 ± 5.29	50.26 ± 6.56	5.779	< 0.001
LVEF	62.17 ± 6.76	51.84 ± 10.02	12.246	< 0.001
Albumin(g/L)	40.54 ± 3.88	39.64 ± 4.00	1.936	0.053
AST	22.00(19.00,27.00)	25.00(20.00,32.50)	3.572	< 0.001
ALT	21.00(16.00,30.00)	29.00(17.50,38.50)	3.358	0.001
Urea	5.81(4.87,6.97)	6.03(5.06,7.24)	1.248	0.212
Creatinine(mg/dl)	0.91 ± 0.95	0.80 ± 0.45	0.132	0.769
eGFR(mL/min/1.73m^2^)	104.03(80.48,123.50)	97.02(79.67,109.70)	1.836	0.066
D-dimer	0.61(0.47,0.75)	0.73(0.53,1.28)	3.581	< 0.001
BNP	106.37(48.40,209.64)	323.13(126.55,861.60)	6.968	< 0.001
CHADS2 score	1.24 ± 1.09	1.93 ± 1.06	5.386	< 0.001
CHA2DS2-VASc score	2.22 ± 1.54	3.33 ± 1.53	6.021	< 0.001
CHA2DS2-VASc score > 2(%)	409(41.8)	49(65.3)	15.670	< 0.001
Baseline pharmacotherapy				
Beta blocker(%)	317(32.41%)	32(42.67%)	3.534	0.06
ACEI/ARB/CCB(%)	528(53.99%)	44(58.67%)	0.872	0.350
Furosemide(%)	159(16.26%)	24(32.00%)	13.456	< 0.001
Antiarrhythmics(%)	214(21.88%)	15(20.00%)	0.072	0.788

*AF* Atrial Fibrillation, *LAAT* Left Atrial Appendage Thrombus, *TIA* Transient Ischemic Attack, *DVT* Deep venous thrombosis, *COPD* Chronic Obstructive Pulmonary Disease, *MPV* Mean platelet volume, *LDL-C* Low-Density Lipoprotein Cholesterol, *LAD* Left atrial diameter, *TAPSE* Tricuspid Annular Plane Systolic Excursion, *LVEDD* Left Ventricular end-diastolic diameter, *LVEF* Left Ventricular Ejection Fraction, *AST* Aspartate Transaminase, *ALT* Alanine Aminotransferase, *eGFR* estimated Glomerular Filtration Rate, *BNP* Brain Natriuretic Peptide, *HF* Heart Failure

**Table 2:** Univariate and multivariate logistic regression analysis for identifying predictors of LAAT

Variable	Univariate logistic		multivariate logistic		AUC (95%CI)
	OR(95%CI)	P	OR(95%CI)	P	
LAD	1.12(1.08,1.16)	< 0.001	1.05(1.00,1.10)	0.049	0.72(0.67, 0.78)
LVEDD	1.13(1.09,1.18)	< 0.001	1.02(0.97,1.07)	0.459	--
LVEF	0.88(0.85,0.90)	< 0.001	0.92(0.89,0.95)	< 0.001	0.21 (0.15, 0.26)
Prior stroke/TIA	2.77(1.42,5.40)	0.003	2.36(1.09,5.10)	0.029	0.55 (0.48, 0.62)
NPAF	6.29(3.28,12.07)	< 0.001	2.35(1.15,4.81)	0.020	0.69 (0.63, 0.74)
Heart failure	8.05(4.73,13.70)	< 0.001	2.99(1.65,5.42)	< 0.001	0.74 (0.68, 0.80)
Creatinine(mg/dl)	2.54(1.35,4.79)	0.004	1.33(0.84,2.10)	0.221	--

Odds ratios are calculated by multivariable logistic analysis

*NPAF* Non-Paroxysmal AF, *CI* Confidence interval, other abbreviations as in Table 1

### Development of the new score system for the predicting LAAT

**Fig. 2:**
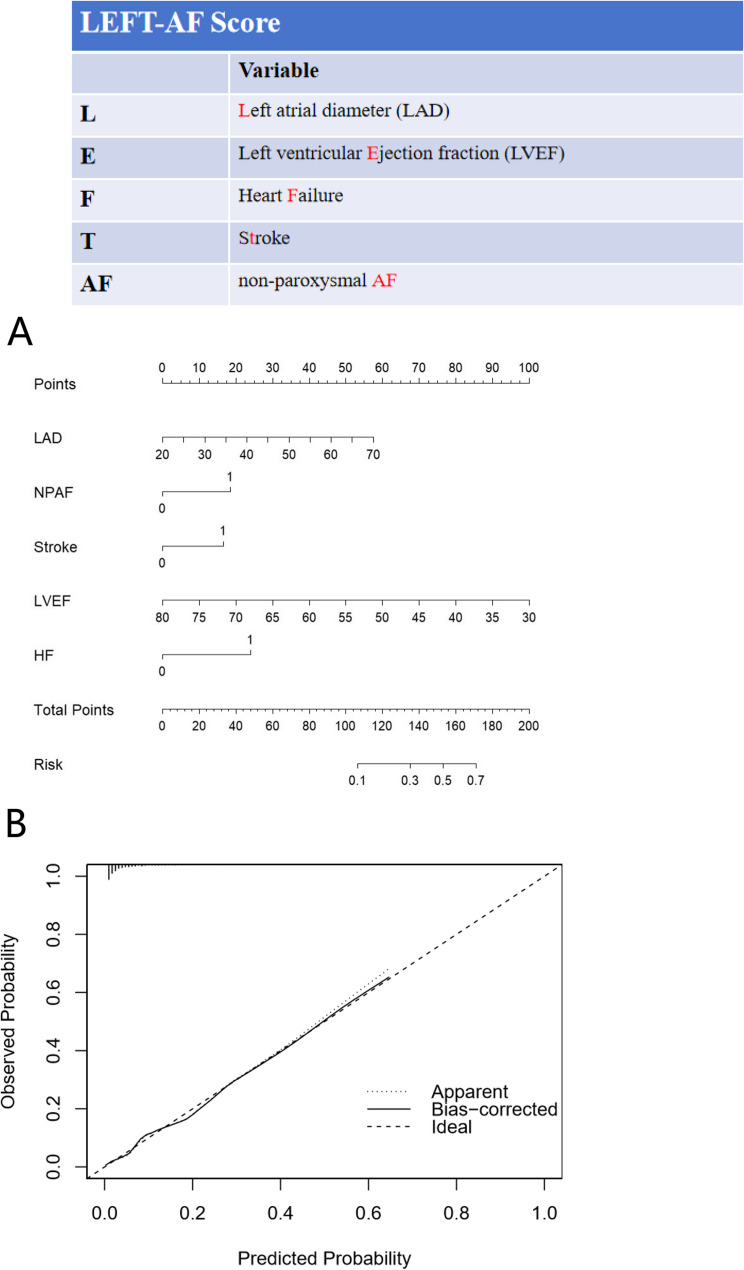
LEFT‐AF risk model. Panel **A**: Nomogram for the risk score. For each predictor, read the points assigned on the 0– 100 scale at the top and then sum these points. Find the number on the ‘Total Points’ scale and then read the corresponding predictions risk of LAAT. Continuous variables are represented from the 1st to the 99th percentiles. The prediction model is preferably used as a web-based calculator or app. Panel **B**: Calibration curve for the risk score. The scattered points in the correction curve do not deviate significantly from the standard line, suggesting that the prediction model shows good accuracy in the derivation cohort

**Fig. 3:**
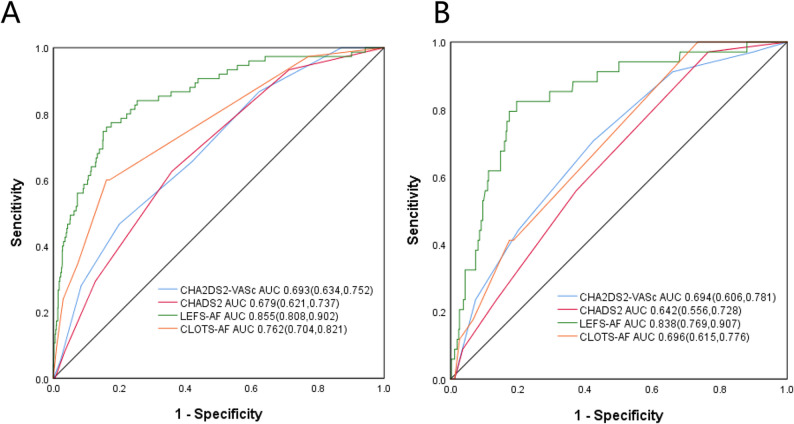
Prediction value of different score systems in the derivation and validation cohorts. Panel **A**, in the derivation cohort, AUC of LEFT-AF risk score compared with CHA2DS2-VASc score, CHADS2 score and CLOTS-AF score. Panel **B**, in the validation cohort, AUC of LEFT-AF risk score compared with CHA2DS2-VASc score, CHADS2 score and CLOTS-AF score. AUC indicates area under the receiver operating characteristic curve

According to the outcomes of the multivariate analysis, the final score was designated as the LEFT-AFscore. This was determined by taking into account factors such as LAD, LVEF, a history of prior stroke, HF, and NPAF (Fig. 2A). The validation model shows a calibration error of 0.005 (Fig. 2B), with the deviation calibration curve closely aligning with the ideal curve. The Hosmer-Lemeshow test indicated that the prediction model exhibits outstanding calibration ability (χ² = 10.395, P = 0.238, df = 8). The AUC values of the LEFT-AF, CHA2DS2-VASc, CHADS2, and CLOTS-AF score systems were 0.855 (95% CI 0.808, 0.902); 0.693 (95% CI 0.634, 0.752); 0.679 (95% CI 0.621, 0.731) and 0.762 (95% CI 0.704, 0.821) (P<0.001), as shown in Fig. 3, panel A.

### Validation of the LEFT-AF score

In the validation cohort,which consisted of 452 patients, 34 (7.52%) patients had experienced of LAAT. The univariate analysis results demonstrated consistent results between the validation and derivation cohorts (Supplemental Table S3). The AUC of the LEFT-AF score for predicting LAAT was 0.838 (95% CI 0.769-0.907), which was higher than that of the CHA2DS2-VASc score (0.694, 95% CI 0.606-0.781) (P<0.001) and the CHADS2 score (0.642, 95% CI 0.556-0.728) (P<0.001), indicating superior predictive performance of the LEFT-AF score (Fig. 3, panel B). The Hosmer-Lemeshow test showed that the prediction model possesses excellent calibration capability (χ2=3.965, P= 0.860, df=8). 

### Thrombus resolution

Throughout the follow-up duration, none of the patients encountered a major bleeding incident. Seven non-major bleeding events occurred, and eighteen patients were lost to follow-up. The rest of the patients adhered to their prescribed anticoagulant treatment plan. Among the LAAT cohort, 84 patients (77%) underwent serial TEE imaging (mean TEE number, 1.7 ± 0.6). The rate of complete thrombus resolution was 71.43%. Baseline characteristics of patients with and without thrombus resolution are presented in Table S3. Thrombus resolution was associated with significantly lower albumin levels (39.15 ± 3.95 vs. 41.54 ± 3.20; *P* = 0.004). Neither clinical and echocardiographic parameters nor the anticoagulation method could predict thrombolysis in this patient group (all *P* > 0.05; Table S3).

## Discussion

Clinically relevant discoveries from this research: (1) to our knowledge, this investigation develops a more accurate and comprehensive risk-stratified scoring system. This system combines various predictors such as clinical factors, biomarkers, and noninvasive echocardiography data to detect patients at high risk of LAAT; (2) LAATs were observed in 7.24% of NVAF patients without anticoagulation therapy; (3) clinical and noninvasive echocardiographic parameters, including LAD, LVEF, history of HF, history of stroke/TIA, NPAF could independently predict LAAT; (4) the LEFT-AF score demonstrates greater predictive accuracy compared to the widely adopted CHA2DS2-VASc and CHADS2 risk stratification models.

### Prevalence of LAAT

Multiple reports within a meta-analysis have detailed the prevalence and features of LAAT in North America and Europe. Among AF patients not undergoing anticoagulation therapy, the prevalence is around 9–27% [21]. Another meta-analysis indicated a weighted mean prevalence of 7.4% for LAAT in studies of patients who did not receive anticoagulant treatment [22]. Schaeffer et al. pointed that the prevalence of LAAT in patients without anticoagulation therapy was 9.5% [23]. In our research, we observed that the prevalence of LAAT in patients not receiving anticoagulant therapy was slightly lower than that reported previously. This discrepancy might be due to the exclusion of SEC patients from the LAAT group. Additionally, gene polymorphism heterogeneity and socioeconomic factors could ultimately influence the development of LAAT in different populations [24].

### Performance of the LEFT-AF score and comparison with existing models

In our research, the CHA2DS2VASc score being ≥ 2 did not turn out to be an independent predictor of LAAT. This implies that its predictive value for LAAT is limited, which is in line with most of the previous studies [3–6, 16, 17]. Recently, an observational research found that LAAT existed in patients with a low CHA2DS2-VASc score, thus there is no correlation between an increased CHA2DS2-VASc score and LAAT [25]. However, we also identified LAAT in 13.3% of the low CHA2DS2-VASc score group.

Several studies have demonstrated that the CHA2DS2-VASc score may predict stroke through an atherosclerotic mechanism [26–28]. Age, sex, and hypertension are risk factors for atherosclerosis, but their ability to independently predict LAAT remains inconsistent despite demonstrating stronger associations with overall stroke risk in AF [25]. Therefore, the CHA2DS2-VASc score may more comprehensively reflect overall stroke risk rather than indicate the risk of LAAT.

In our study, despite a higher prevalence of HF, stroke, and vascular disease in patients with LAAT, a CHA2DS2-VASc score ≥ 2 did not show a significant difference between the two groups. This aligns with prior observations that not all factors in the CHA2DS2-VASc score confer equivalent risk due to the variations in both temporal exposure and clinical severity of underlying conditions [29]. This may partly illustrate modest predictive capacity of the CHA2DS2-VASc score for LAAT risk prediction in the present and prior studies.

Our newly developed LEFT-AF score, which integrates clinical factors, biomarkers, and noninvasive echocardiography data, demonstrates superior predictive accuracy for LAAT compared to the widely adopted CHA2DS2-VASc and CHADS2 scores. Importantly, when compared to the recently published CLOTS-AF score [16], which also aims to predict LAAT, the LEFT-AF score demonstrates better predictive ability. ROC curve comparisons using the DeLong test indicate that the AUC of the LEFT-AF score is substantially higher than that of the CLOTS-AF score (AUC: 0.838 vs. 0.696), the CHA2DS2-VASc score (AUC: 0.85 vs. 0.694), and the CHADS2 score (AUC: 0.85 vs. 0.642), with all p-values less than 0.001, indicating that the LEFT-AF model provides a meaningful advancement in risk stratification. This enhanced performance is likely attributable to the comprehensive inclusion of robust echocardiographic parameters like LAD and LVEF, and the refinement of clinical risk factors such as heart failure severity and AF type, offering a more nuanced and accurate tool for identifying high-risk patients.

### Additional predictors

#### AF type

The current ESC guidelines do not include AF type or AF burden as determinants of LAAT formation, but several studies have showen that non-paroxysmal AF confers a greater LAAT risk than paroxysmal AF [13, 17, 30, 31]. A meta-analysis also confirmed a significant association between NPAF and an increased risk of thromboembolism, highlighting the necessity for further investigation to incorporate different types of AF into models assessing thromboembolism risk [32]. Meanwhile, further investigation is needed on the role of AF type in the risk prediction model.

#### Cardiac remodeling

Our multivariate analysis identified reduced LVEF as a robust independent determinant of LAAT formation in atrial fibrillation patients, consistent with prior reports establishing this association [33, 34]. Additionally, one study demonstrated that the relative risk of LAAT increases 110% for every 10% reduction in LVEF [35]. Melduni et al. also showed that LVEF is the strongest predictive value for LAAT (AUC = 0.78), with LVEF ≤ 40% showing 62% sensitivity and 75% specificity in detecting LAAT [36]. Therefore, considering that different patients may present with varying degrees of HF, it seems unreasonable to assign a fixed score for HF patients in the CHA2DS2-VASc scoring system. It is important to consider the varying weight of different indicators and further refine the scoring system according to ejection fraction in order to more accurately reflect the impact of HF on the overall score.

Previous research has demonstrated that Left atrial (LA) enlargement, another indicator of heart remodeling, is also a significant independent predictor of LAAT [37, 38]. Herring et al. found that in patients receiving continuous warfarin therapy, LAD >46 cm and CHA2DS2-VASc score ≥ 1 could identify 91.5% of those at risk of developing LAAT [39]. These results, which align with ours, indicate that LA enlargement could predict LAAT in patients with NVAF. These results can be corroborated by the work of Goldman et al., who proposed that LA enlargement results in reduced LA emptying, turbulent blood flow, and subsequent formation of LAAT due to LA blood stasis [40]. Iwakura et al. have also reported that decreased LVEF and elevated LV filling pressure contribute to LA dilatation and the formation of LAAT [41]. Similarly, Ayirala et al. have found that LVEF and LA volume are independent predictors of LAAT diagnosed by TEE [42]. There is increasing evidence that echocardiographic assessment of the left ventricular and atrial structure and function significantly improves LAAT risk prediction compared to clinical features alone.

### Thrombus resolution

Resolution of LAAT was observed in 71.43% of the 84 patients who underwent repeated TEE imaging for the purpose of cardioversion or catheter ablation (CA). Previous observational studies have reported varying rates of LAAT resolution, ranging from 50% to 90% [43, 44]. In our research, the exploratory analysis revealed that low albumin levels were the only predictors of LAAT, rather than LVEF, NPAF, and LA size as previously reported [45, 46]. Due to the limitations of its small sample size and insufficient statistical power, these findings need to be verified in larger-scale prospective studies. We hypothesize that reducted serum albumin levels in these patients may decrease the binding affinity for anticoagulant drugs, leading to elevated blood drug concentrations and consequently increased thrombolysis rate.

### Clinical implications

Both our investigation and prior large-scale analyses have consistently demonstrated that NVAF patients exhibit LAAT risk despite falling into the low-risk CHA2DS2-VASc category. However, this patient population maintains substantial stroke risk, approximately 10%, due to LAAT [46]. Risk stratification models incorporating LAAT predictors may improve patient selection.

This study aims to develop and validate a convenient thrombosis risk prediction tool applicable to the initial clinical assessment of patients with atrial fibrillation. We intend to limit the predictive variables to demographic information, clinical characteristics, and transthoracic echocardiographic parameters, which can be routinely obtained before the patient is referred for transesophageal echocardiography. We recognize that left atrial flow velocity and morphology, as evaluated by transesophageal echocardiography, are well-established parameters for assessing thrombosis risk [47, 48]. Nevertheless, a model that depends on transesophageal echocardiography (TEE) parameters to determine the necessity of TEE gives rise to a logical loop in clinical practice, thereby restricting its application. Consequently, the distinctive value of this model resides in its “pre-TEE” utilization, which can offer clinicians a prompt and efficient preliminary risk assessment prior to contemplating a semi-invasive examination. Future investigations could explore the integration of this score with TEE parameters to perform more refined risk stratification for specific high-risk populations.

### Study limitations

This was a two-center retrospective analysis of LAAT incidence, which may have site-specific bias. (1) Despite our relatively large sample size, the number of LAAT events was somewhat limited due to the exclusion of SEC in the analysis of the thrombus group. Although there are studies demonstrating a clear correlation between SEC and thrombosis, we believe that excluding SEC can better reflect the actual incidence of LAAT; (2) The study population specifically involved TEE patients with NVAF who had not received appropriate anticoagulation therapy prior to cardioversion or ablation. Consequently, these findings may have limited generalizability to the broader NVAF population. 3.We exclusively considered parameters of TTE mentioned in previous studies that could potentially be used to predict LAAT, while other unaccounted parameters may also exist. 4. While we categorized atrial fibrillation types, the absence of continuous arrhythmia duration data precluded precise quantification of cumulative AF burden in NVAF. 5. The research participants were all recruited from the eastern region of China. Given the disparities in genetic background, clinical practices, and lifestyle among individuals of diverse races and regions, the predictive model derived from this study has not been conclusively shown to be universally applicable when extrapolated to other populations. Thus, prior to its extensive application, it is imperative to perform external validation in large-scale prospective cohorts encompassing other regions of China and different races to further verify its effectiveness and evaluate the calibration requirements. 6. It is particularly noteworthy that this exploratory analysis of thrombolysis predictors, especially within the low albumin subgroup, involves a relatively small sample size. Consequently, the findings should be considered preliminary and hypothesis-generating, and further validation in larger prospective cohorts is also necessary.

## Conclusions

The novel LEFT-AF scoring system may help improve the risk prediction ability of LAAT and facilitate the identification of high-risk patients for LAAT, particularly in patients stratified as low-to-intermediate stroke risk (CHA2DS2-VASc score < 2) for whom current guidelines do not suggest routine anticoagulation. The predictive accuracy and clinical utility of our novel composite scoring system for LAAT in NVAF needs further validation.

## Supplementary Information

Supplementary Material 1.

## Data Availability

The data underlying this article will be shared on reasonable request to the corresponding author.

## Abbreviations

AF: Atrial Fibrillation
NVAF: Nonvalvular atrial fibrillation
LAAT: Left Atrial Appendage Thrombus
NPAF: Non-Paroxysmal Atrial Fibrillation
TIA: Transient Ischemic Attack
DVT: Deep venous thrombosis
COPD: Chronic Obstructive Pulmonary Disease
LAD: Left atrial diameter
TAPSE: Tricuspid Annular Plane Systolic Excursion
LVEDD: Left Ventricular end-diastolic diameter
LVEF: Left Ventricular Ejection Fraction
HF: Heart Failure
TEE: Transesophageal echocardiography
