# Supplementary material for: A new risk-stratified scoring system for predicting left atrial appendage thrombus in patients with nonvalvular atrial fibrillation
Source: BMC Cardiovasc Disord. 2025 Dec 20;26:72. doi: 10.1186/s12872-025-05348-6 (PMC12837345; doi:10.1186/s12872-025-05348-6)
Supplement: Supplementary file 1 — Supplementary Material 1. [file 12872_2025_5348_MOESM1_ESM.doc]

Supplemental Material

**Figure S1. Distribution of the patients and frequency of the LAAT detection by TEE in each CHA2DS2-VASc score.**

**
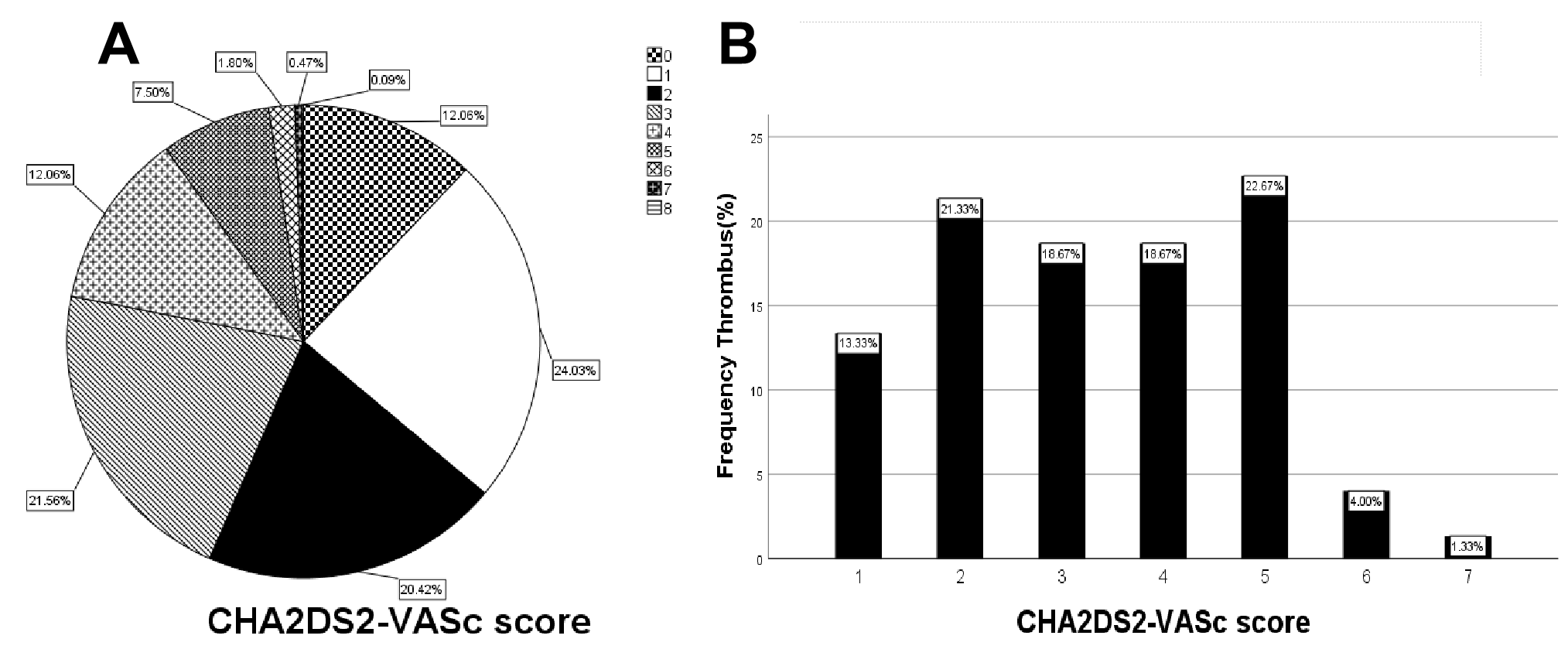
**

A:Among 1053 AF patients, 127 (12.06%) had a CHA2DS2-VASc score of 0, 253 (24.03%) had 1, 215 (20.4%) had 2, 227 (21.56%) had 3, 127 (12.06%) had 4, 79 (7.5%) had 5, 19 (1.8%) had 6, 5 (0.47%) had 7,and 1 (0.09%) had 8. B: The numbers of patients with LAAT in a CHA2DS2-VASc score of 1 was 13.33% (n=10), 2 was 21.33% (n=16), 3 was 18.67% (n=14), 4 was 18.67% (n=14), 5 was 22.67% (n=17), 6 was 4.00% (n=3), and 7 was 1.33% (n=1). No patients with a CHA2DS2-VASc score of 0 and 8 had LAAT.

**FigureS 2. The Lasso regression model constructed.**

**A B**


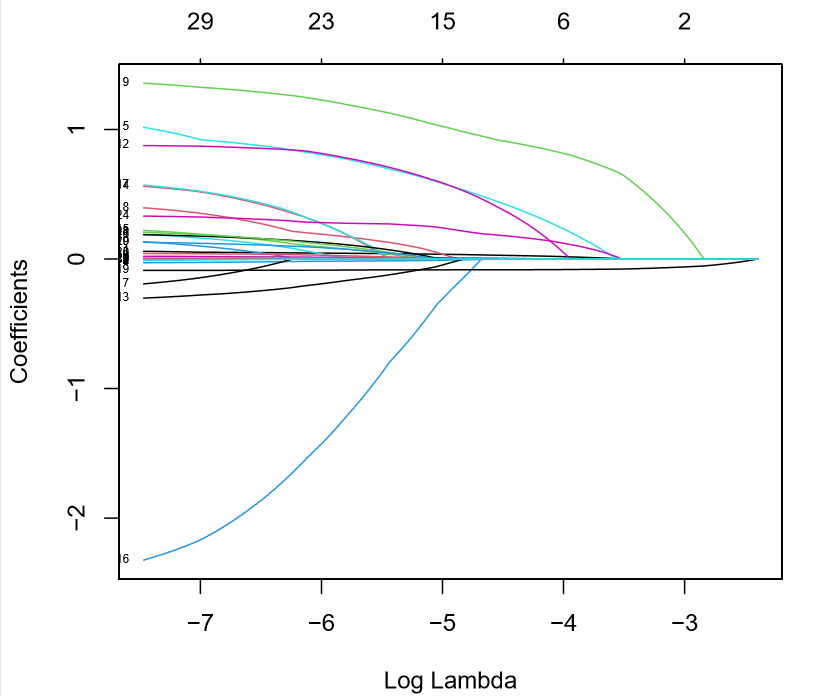

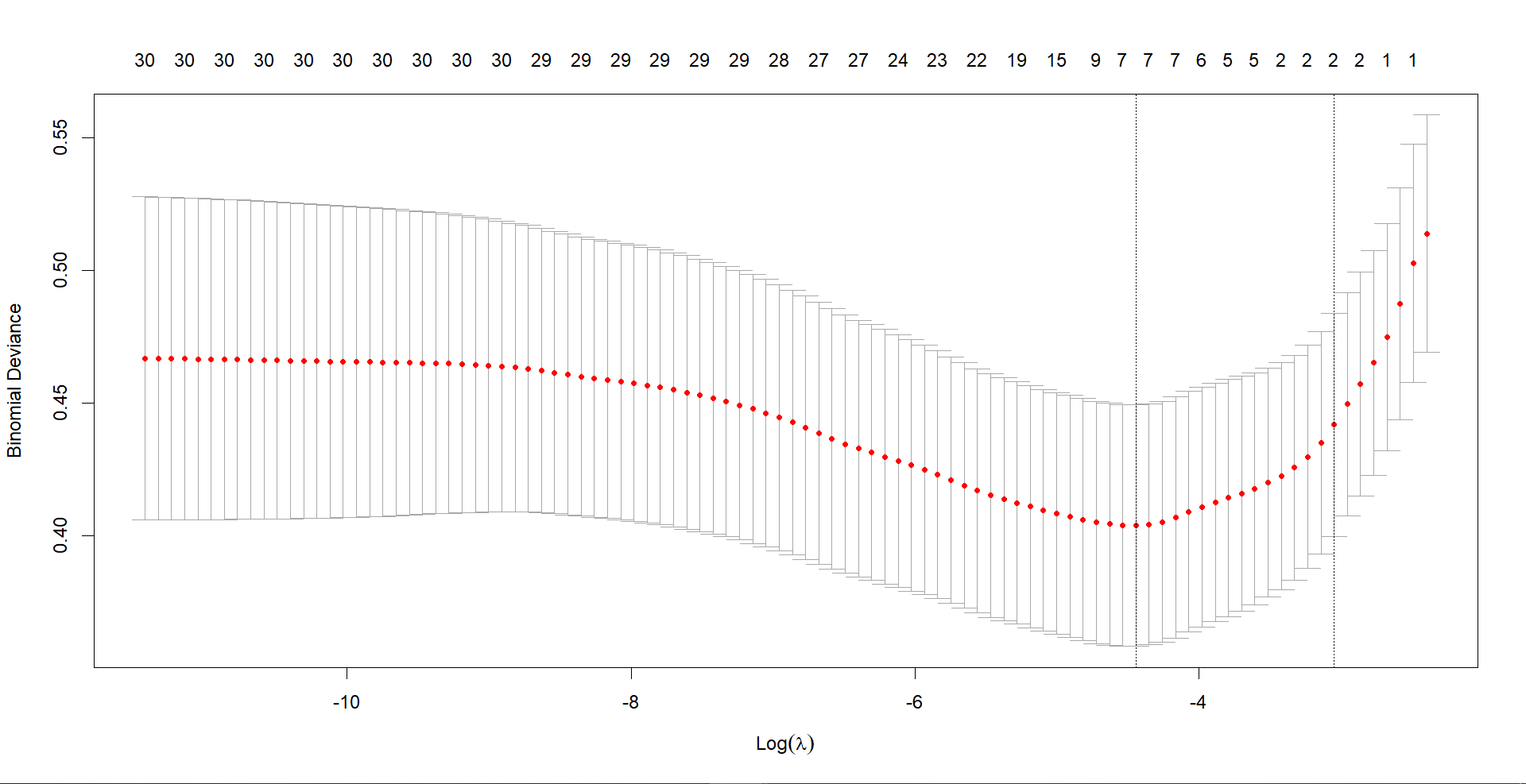


1. Lasso regression model was constructed to reduce clinically relevant collinearity.

**B**. 7 indicators are considered relatively optima.

**Table S1. Baseline characteristics of patients in derivation and validation cohorts.**

| Variable | Derivation cohort（n=1053） | Validation cohort（n=452） | χ^2^/t/z | P |
| --- | --- | --- | --- | --- |
| Gender |  |  | 0.606 | 0.436 |
| Female (%) | 420(39.9) | 190(42.0) |  |  |
| Male (%) | 633(60.1) | 262(58.0) |  |  |
| Age (years） | 63.38±9.69 | 63.37±9.94 | 0.024 | 0.981 |
| Weight(Kg) | 75.66±12.85 | 74.57±12.39 | 1.440 | 0.150 |
| Type of AF |  |  | 0.031 | 0.861 |
| Paroxysmal AF(%) | 519(49.3) | 225(49.8) |  |  |
| Non-Paroxysmal AF(%) | 534(50.7) | 227(50.2) |  |  |
| Patients with/without LAA(%) | 75（7.12%） | 34（7.52%） | 0.733 | 0.392 |
| Duration of Disease（month） | 12.00(2.00,60.00) | 24.00(3.00,60.00) | 0.657 | 0.511 |
| Drinking(%) | 322(30.6) | 139(30.8) | 0.004 | 0.947 |
| Smoking(%) | 380(36.1) | 149(33.0) | 1.353 | 0.245 |
| Heart failure(%) | 304(28.9) | 137(30.3) | 0.316 | 0.574 |
| Hypertension(%) | 581(55.2) | 251(55.5) | 0.016 | 0.899 |
| Diabetes(%) | 211(20.0) | 105(23.2) | 1.943 | 0.163 |
| Prior stroke/TIA(%) | 75(7.1) | 27(6.0) | 0.661 | 0.416 |
| Coronary heart disease(%) | 228(21.7) | 93(20.6) | 0.219 | 0.640 |
| Other arterial embolism(%) | 23(2.2) | 9(2.0) | 0.057 | 0.812 |
| DVT(%) | 11(1.0) | 7(1.5) | 0.680 | 0.410 |
| COPD(%) | 13(1.2) | 6(1.3) | 0.022 | 0.882 |
| MPV | 10.06±0.97 | 10.10±0.95 | 0.609 | 0.542 |
| LDL-C | 2.91±1.07 | 2.89±0.97 | 0.132 | 0.895 |
| LAD | 41.50±6.49 | 41.43±6.66 | 0.185 | 0.853 |
| TAPSE | 21.65±3.33 | 21.70±3.01 | 0.287 | 0.774 |
| LVEDD | 46.10±5.51 | 46.15±6.06 | 0.164 | 0.870 |
| LVEF | 61.44±7.52 | 61.02±7.99 | 0.979 | 0.328 |
| Albumin(g/L) | 40.48±3.89 | 40.51±4.23 | 0.110 | 0.912 |
| AST | 22.00(19.00,27.00) | 22.00(18.00,29.00) | 0.003 | 0.998 |
| ALT | 21.00(16.00,31.00) | 20.00(15.00,32.00) | 0.799 | 0.424 |
| Urea | 5.81(4.90,6.97) | 5.74(4.75,6.82) | 1.259 | 0.208 |
| Creatinine(mg/dl) | 0.78±0.37 | 0.78±0.47 | 1.310 | 0.190 |
| eGFR(mL/min/1.73m^2)^ | 103.53(80.45,123.00) | 103.62(81.66,121.64) | 0.169 | 0.866 |
| D-dimer | 0.61(0.47,0.78) | 0.60(0.48,0.84) | 0.410 | 0.682 |
| BNP | 112.24(52.45,233.96) | 131.90(55.07,266.39) | 1.463 | 0.143 |
| CHADS2 score | 1.29±1.10 | 1.36±1.09 | 1.299 | 0.194 |
| CHA2DS2-VASc score | 2.30±1.57 | 2.36±1.49 | 0.676 | 0.499 |
| CHA2DS2-VASc score>2(%) | 458(43.5) | 200(44.2) | 0.073 | 0.787 |
| **Baseline pharmacotherapy** | | | | |
| Beta blocker(%) | 324(30.77%) | 153(33.85%) | 1.124 | 0.289 |
| ACEI/ARB/CCB(%) | 588(55.84%) | 244(53.86%) | 0.723 | 0.395 |
| Furosemide(%) | 169(16.05%) | 75(16.56%) | 0.035 | 0.851 |
| Antiarrhythmics(%) | 232(22.03%) | 90(19.91%) | 1.012 | 0.315 |

AF, Atrial Fibrillation; LAAT, Left Atrial Appendage Thrombus; TIA, Transient Ischemic Attack; DVT, Deep venous thrombosis; COPD, Chronic Obstructive Pulmonary Disease; MPV, Mean platelet volume; LDL-C, Low-Density Lipoprotein Cholesterol; LAD, Left atrial diameter; TAPSE, Tricuspid Annular Plane Systolic Excursion; LVEDD, Left Ventricular end-diastolic diameter; LVEF, Left Ventricular Ejection Fraction; AST, Aspartate Transaminase; ALT, Alanine Aminotransferase; eGFR,estimated Glomerular Filtration Rate; BNP, Brain Natriuretic Peptide; HF, Heart Failure.

**Table S2. Comparison of the parameters between patients with and without LAAT in the validation cohort.**

| Variable | NO LAAT (n=418) | LAAT (n=34) | χ^2^/t/z | P |
| --- | --- | --- | --- | --- |
| Gender |  |  | 2.405 | 0.121 |
| Female (%) | 180 (43.1) | 10 (29.4) |  |  |
| Male (%) | 238 (56.9) | 24 (70.6) |  |  |
| Age (years) | 63.39±9.90 | 63.21±10.59 | 0.101 | 0.920 |
| Type of AF |  |  | 15.184 | <0.001 |
| Paroxysmal AF (%) | 219 (52.4) | 6 (17.6) |  |  |
| Non-Paroxysmal AF (%) | 199 (47.6) | 28 (82.4) |  |  |
| Drinking (%) | 129 (30.9) | 10 (29.4) | 0.031 | 0.860 |
| Smoking (%) | 137 (32.8) | 12 (35.3) | 0.090 | 0.764 |
| Heart failure (%) | 110 (26.3) | 27 (79.4) | 41.965 | <0.001 |
| Hypertension (%) | 233 (55.7) | 18 (52.9) | 1.000 | 0.752 |
| Diabetes (%) | 98 (23.4) | 7 (20.6) | 0.144 | 0.704 |
| Prior stroke/TIA (%) | 25 (6.0) | 2 (5.9) | 0.001 | 0.981 |
| Coronary heart disease (%) | 86 (20.6) | 7 (20.6) | <0.001 | 0.998 |
| Other arterial embolism (%) | 7 (1.7) | 2 (5.9) | -- | 0.142 |
| DVT (%) | 4 (1.4) | 1 (2.9) | -- | 0.424 |
| COPD (%) | 5 (1.5) | 1 (2.9) | -- | 0.376 |
| LAD, mm | 41.10±6.62 | 45.47±5.85 | 3.732 | <0.001 |
| TAPSE, mm | 21.78±2.97 | 20.69±3.25 | 2.038 | 0.042 |
| LVEDD, mm | 45.95±5.88 | 48.67±7.60 | 2.538 | 0.011 |
| LVEF, % | 61.59±7.48 | 53.97±10.55 | 5.515 | <0.001 |
| Albumin, (g/L) | 40.61±4.26 | 39.18±3.67 | 1.909 | 0.057 |
| AST, u/L | 22.00 (18.00, 29.00) | 25.00 (21.00, 40.00) | 2.842 | 0.004 |
| ALT, u/L | 20.00 (15.00, 32.00) | 22.50 (17.00, 35.00) | 1.776 | 0.076 |
| Urea, mmol/L | 5.73 (4.75, 6.75) | 6.40 (5.05, 7.49) | 1.552 | 0.121 |
| Creatinine, mg/dl | 0.92±0.93 | 0.85±0.34 | 0.124 | 0.873 |
| eGFR(mL/min/1.73m^2^) | 103.98(81.69,121.97) | 97.49(79.04,112.17) | 0.918 | 0.556 |
| D-dimer, mg/L | 0.60 (0.48, 0.80) | 0.83 (0.50, 1.18) | 2.503 | 0.012 |
| BNP, ng/L | 119.22 (51.38, 236.92) | 315.99 (159.54, 713.50) | 5.048 | <0.001 |
| CHA2DS2-VASc score | 2.28±1.48 | 3.29±1.40 | 3.851 | <0.001 |
| CHA2DS2-VASc score>2 (%) | 176 (42.1) | 24 (70.6) | 10.340 | 0.001 |
| **Baseline pharmacotherapy** |  |  |  |  |
| Beta blocker (%) | 122 (29.19%) | 16 (47.06%) | 5.670 | 0.017 |
| ACEI/ARB/CCB (%) | 262 (62.68%) | 18 (52.94%) | 0.593 | 0.441 |
| Furosemide (%) | 60 (14.35%) | 11 (32.35%) | 8.636 | 0.003 |
| Antiarrhythmics (%) | 96 (22.97%) | 7 (20.59%) | 0.030 | 0.862 |

Abbreviations as in Table S1

**Table S3. Characteristics of cohorts according to thrombus resolution status.**

| Variable | Thrombus resolution（n=60） | No resolution（n=24） | χ2/t/z | P |
| --- | --- | --- | --- | --- |
| Gender |  |  | 0.373 | 0.541 |
| Female (%) | 16(26.67) | 8(33.33) |  |  |
| Male (%) | 44(73.33) | 16(66.67) |  |  |
| Age (years） | 63.44±9.76 | 62.66±9.77 | 1.372 | 0.172 |
| Weight(Kg) | 75.99±13.32 | 81.60±11.94 | 1.987 | 0.047 |
| Type of AF |  |  | 0.212 | 0.645 |
| Paroxysmal AF(%) | 9(15.0) | 2(8.33) |  |  |
| Non-Paroxysmal AF(%) | 51(85.0) | 22(91.67) |  |  |
| Anticoagulation time（day） | 78.0(58.0,93.0) | 76.0(59.0,101.0) | 0.114 | 0.912 |
| Drinking(%) | 20(33.33) | 5(20.83) | 1.281 | 0.258 |
| Smoking(%) | 23(38.33) | 5(20.83) | 2.363 | 0.124 |
| Heart failure(%) | 41(68.33) | 18(75.00) | 0.364 | 0.546 |
| Hypertension(%) | 30(50.0) | 15(62.50) | 1.077 | 0.299 |
| Diabetes(%) | 13(21.67) | 4(16.67) | 0.046 | 0.830 |
| Prior stroke/TIA(%) | 8(13.33) | 2(8.33) | 0.071 | 0.790 |
| Coronary heart disease(%) | 12(20.00) | 4(16.67) | 0.002 | 0.965 |
| Other arterial embolism(%) | 5(8.33) | 0(0) | 0.898 | 0.343 |
| DVT(%) | 2(3.33) | 0(0) | 0.013 | 0.910 |
| COPD(%) | 2(3.33) | 1(4.17) | 0.000 | 1.000 |
| MPV | 10.17±0.89 | 10.38±0.85 | 0.560 | 0.579 |
| LDL-C | 2.75±1.06 | 3.19±0.740 | 2.154 | 0.031 |
| LAD | 44.83±5.44 | 46.20±5.43 | 0.609 | 0.546 |
| TAPSE | 20.31±4.58 | 20.71±3.42 | 0.022 | 0.985 |
| LVEDD | 48.82±6.02 | 48.43±7.09 | 0.585 | 0.563 |
| LVEF | 53.20±11.48 | 54.33±8.86 | 0.159 | 0.877 |
| Albumin(g/L) | 39.15±3.95 | 41.54±3.20 | 2.817 | 0.004 |
| AST | 25.00(21.00,35.75) | 24.00(18.25,32.00) | 1.071 | 0.288 |
| ALT | 28.50(17.00,40.50) | 24.00(15.25,34.00) | 1.169 | 0.245 |
| Urea | 6.26(4.92,8.27) | 5.66(5.05,6.58) | 1.238 | 0.218 |
| Creatinine(mg/dl) | 0.94±0.94 | 0.82±0.18 | 0.116 | 0.908 |
| eGFR(mL/min/1.73m^2^) | 97.49(79.49,108.62) | 108.91(92.55,125.98) | 1.852 | 0.065 |
| D-dimer | 0.73(0.52,1.18) | 0.69(0.49,1.16) | 0.381 | 0.707 |
| BNP | 326.00(140.21,859.12) | 303.39(141.91,515.57) | 0.342 | 0.738 |
| CHADS2 score | 1.88±1.166 | 1.83±0.702 | 0.000 | 1.000 |
| CHA2DS2-VASc score | 3.35±1.706 | 3.33±1.31 | 0.096 | 0.926 |
| CHA2DS2-VASc score>2(%) | 40(66.67) | 18(75.00) | 0.557 | 0.455 |
| **Baseline pharmacotherapy** | | | | |
| Beta blocker(%) | 27(45.00%) | 11(45.83%) | 0.005 | 0.945 |
| ACEI/ARB/CCB(%) | 30(50.00%) | 17(70.83%) | 3.019 | 0.082 |
| Furosemide(%) | 21(35.00%) | 5(20.83%) | 1.610 | 0.205 |

Abbreviations as in Table S1.
